# Supplementary material for: Validity and Reliability of Questionnaires That Assess Barriers and Facilitators of Sedentary Behavior in the Pediatric Population: A Systematic Review
Source: Int J Environ Res Public Health. 2022 Dec 15;19(24):16834. doi: 10.3390/ijerph192416834 (PMC9778761; doi:10.3390/ijerph192416834)
Supplement: Supplementary file 1 [file ijerph-19-16834-s001.zip › ijerph-2036085-supplementary.pdf]

Supplementary Table S1: Questions about barriers and facilitators of sedentary behavior from the questionnaires evaluated.

First Author	Questions	Answer options
Jago et al, 2005 [18]	**Questionnaire not reported**	
Ridley et al, 2006 [21] (Multimedia Activity Recall for Children and Adolescents - MARCA)	**Questionnaire available**	
Jago et al, 2009 [19] (Questionnaire Physical Activity Self-Efficacy - PASE)	How sure are you that you have (can)…	limit watching TV to 1 hour at least one school day?<br>limit watching TV to 1 hour per day for most school days?<br>not watch TV at all for one school day?<br>limit watching TV to 1 hour at least one non-school day, including the weekend?<br>limit watching TV to 1 hour most non-school days, including weekend?<br>not watch TV at all for most school days?<br>not watch TV at all for one non-school day, including the weekend?<br>not watch TV at all for most non-school days, including weekend?<br>limit playing computer or video games to 1 hour at least one school day?<br>not play computer or video games at all for one school day?<br>limit playing computer or video games to 1 hour for most school days?<br>limit playing computer or video games to 1 hour at least 1 non-school day, including the weekend?<br>limit playing computer or video games to 1 hour for most non-school days, including the weekend?<br>not play computer or video games at all for most school days?<br>not play computer or video games at all for one non-school day, including the weekend?<br>not play computer or video games at all for most non-school days,

		including the weekend?<br>limit talking on the telephone to 1 hour at least one school day?<br>limit talking on the telephone to 1 hour for most school days?<br>limit talking on the telephone to 1 hour for most non-school days, including the weekend?<br>limit talking on the telephone to 1 hour at least one non-school day, including the weekend?<br>not talk on the telephone at all for one school day?<br>not talk on the telephone at all for most school days?<br>not talk on the telephone at all for one non-school day, including the weekend?<br>not talk on the telephone at all for most non-school days, including weekend?
**Author**	**Questions**	**Scale the Likert**
Dwyer et al, 2011 [21] (Preschool-age Children's Physical Activity Questionnaire - Pre-PAQ)	How much do you agree with the following statements?	I am physically active with or in front of my child<br>I limit what my child does as I worry that he/she may injury themselves<br>I focus upon my child developing their basic learning skills such as numbers and letters.<br>My work schedule or other commitments limit the time I have to play with my child.
	How well do these statements describe your child?<br>(Please tick one box and one response for each statement)	My child needs me to<br>motivate him/her to play<br>My child needs company (e.g., friends, siblings, parents, adults) to
**Author**	**Questions**	**Answer options**

	**Controlling Practices around Physical Activity**	**No access to answer options**
	Rules around active play indoors	How often is your child allowed to do each of the following activities while playing inside your house…<br>…climbing<br>…hopping, skipping or galloping
	Rules around active play outdoors	How often do you ask your child…<br>…to calm down his/her outdoor play?<br>…not to run while s/he is playing outside?
	Use of PA to reward/control behavior	How often does your child get extra outside time as a reward?<br>How often do you offer sports or physical activities to your child as a reward for good behavior?
	**Controlling Practices around Screen Time**	**No access to answer options**
Vaughn et al. 2013 [16] (Quesitonnaire's name not reported)	Explicit modeling and enjoyment of PA	…hear you talk about participating in a sport or being physically active?<br>…see you doing, or going to do, something that is physically active?
	Verbal encouragement for PA	During a typical week, how often do you…<br>…say things to encourage your child to spend less time being sedentary?<br>…tell your child how sedentary habits can be unhealthy?
	Logistic support for sports	How active are you in enrolling your child in sports?<br>For how many activities have you or other adult paid fees?
	Logistic support for active play	During a typical week, how often do you take your child to the park to play?<br>During the last month, how many times have you taken your child to play at a park?
	Importance and value of PA	How important is it for your child to be physically active when s/he grows up?<br>How valuable is it to you that your child be physically active?
	Support/reinforcement from other adults	Other adults in my child's life make it hard to…<br>…get my child to be physically active.<br>…enforce household rules about TV viewing.
	Limiting or monitoring screen time	About how much time is s/he allowed to watch TV, videos, or movies each weekday/ weekend day?

	Use of screen time to reward/control behavior	How often do you…<br>…use TV time to control your child's behavior?<br>…take away TV, video, or movie time as a punishment for bad behavior?
		During a typical week, how often do you watch TV or videos with your child?<br>I enjoy watching TV/movies with my child.
	Exposure to TV	How many days per week does your family have the television on during…<br>…breakfast?<br>…the evening meal?
	Explicit modeling and enjoyment of screen time	During a typical week, how often do you watch TV or videos with your child?<br>I enjoy watching TV/movies with my child.
	Limiting outdoor play due to weather	How often do you let your child play outside…<br>…on cold days?<br>…on hot days?
**Author**	**Questions**	**Multiple Answer**
Norman et al, 2018 [20] (Eating and Physical Activity Questionnaire - EPAQ)	Items (How certain are you that you can . . .)	Get your child to be physically active outdoors several times a week for example by structured play?<br>Get your child to participate in organized physical activity at least once a week, for example by dancing, swimming, or playing soccer?<br>Get your child to be physically active (e.g., playing outdoors) instead of watching TV or playing computer games?<br>Walk with your child to and from school several times a week?<br>Be a good role model by being physically active yourself several times a week, for example, by participating in sports, swimming, or bike riding?
	Items (How certain are you that you can . . .)	Limit your child to watch TV, DVDs, or play on the computer, smartphone or tablet 2 hours a day at the most?
**Author**	**Questions**	**Scale the Likert**
Dwyer et al, 2011	How much do you agree with the following	Encourage my child to play outside when

	statements?	the weather is suitable
(Pre-PAQ)<br><br>Questionnaire	How well do these statements describe your child?<br>(Please tick one box and one response for each statement)	It is safe for my child to play outdoors in<br><br>my neighborhood (if supervised)<br><br>There are usable footpaths on most of the streets<br>in my local area<br>There are major barriers or dangers to walking<br>with my child in my neighborhood that make it hard to get from place to place (for example, major roads, railway lines, canals, storm water drains or rivers).<br>There is so much traffic along the streets that it<br>makes it difficult or dangerous to walk with my child in my neighborhood<br>There are sufficient traffic lights or pedestrian<br>crossings to make it safe to walk with my child around my neighborhood.<br>The level of crime in my neighborhood makes<br>it unsafe to go on walks with my child during the day<br>The local shop(s) are within easy walking distance<br>of my home<br>There are dangers (e.g. dogs, undesirable people) in<br>the local park(s) so I avoid taking my child there.<br>My child has a very active<br>nature
	How often does your child use the facilities listed below to play and be physically activity, in a typical month, when the weather is suitable?<br>(please tick as many responses as apply)	Open areas such as beaches, rivers, natural reserves<br><br>Park or oval<br>Public playground<br>Swimming pool (public or private)
		**Multiple Answer**
	What best describes your backyard?<br>(please tick *one response*)	No yard at all<br>No private yard

		A small yard<br>A medium yard (eg. a standard block of land)<br>A large yard (eg. ¼ acre/1000m2 or more)
	What was the weather like YESTERDAY?<br>(please tick one response)	Fine to play outdoors<br>Too wet to play outdoors<br>Too hot or humid to play outdoors<br>Too cold to play outdoors
	Does your local neighbourhood have the following<br>places or facilities where your child can be play<br>and be physically active? (please tick as many<br>responses as apply)	Open areas such as beaches, rivers, natural reserves<br>Public park or oval<br>Playground<br>Public swimming pool<br>Gym that offers programs for young children e.g. kindergym, playgym etc.<br>Club that offers activities/sports for young children e.g. soccer, dance etc.

Author	Questions	Scale the Likert
Fillon et al. 2022 [22] (Physical and Sedentary Activity Questionnaire for Children and Adolescents - CAPAS-Q)	On average, how much time do you spend sitting each day in class, at recess,<br>canteen, etc.	Less than 2 hours<br>2 am to 4 am<br>4 am to 6 am<br>6am to 8am<br>8am to 10am<br>More than 10 am
	On average, on weekends or holidays, how much time do you spend<br>day in front of a screen (computer, tablet, phone, video game, TV, etc.)?	Less than 2 hours<br>2 am to 4 am<br>4 am to 6 am<br>6am to 8am<br>8am to 10am

		More than 10 am
	On school days, how many times a day do you sit for more than 1.5 hours without moving for at least 2 minutes straight?	0 times<br>1 time<br>2 time<br>3 time<br>4 time<br>more than 4 times
